# Antimalarial activity of Malaria Box Compounds against *Plasmodium falciparum* clinical isolates

**DOI:** 10.1016/j.ijpddr.2017.10.005

**Published:** 2017-10-16

**Authors:** Jersley D. Chirawurah, Felix Ansah, Prince B. Nyarko, Samuel Duodu, Yaw Aniweh, Gordon A. Awandare

**Affiliations:** West African Center for Cell Biology of Infectious Pathogens and Department of Biochemistry, Cell and Molecular Biology, University of Ghana, P. O. Box LG 54, Volta Road Legon, Accra, Ghana

**Keywords:** *Plasmodium*, Malaria box, Clinical isolates, Potency, Erythrocytes, Compounds

## Abstract

Malaria remains a major cause of childhood deaths in resource-limited settings. In the absence of an effective vaccine, drugs and other interventions have played very significant roles in combating the scourge of malaria. The recent reports of resistance to artemisinin necessitate the need for new antimalarial drugs with novel mechanisms of action. Towards the development of new, affordable and easily accessible antimalarial drugs for endemic regions, the Medicines for Malaria Venture (MMV) assembled a total of 400 active antimalarial compounds called the Malaria Box. The potency and the efficacy of the Malaria Box Compounds have been determined mainly using laboratory strains of *P. falciparum*.

This study investigated the potency of twenty compounds from the Malaria Box against four clinical isolates from Ghana, using optimized *in vitro* growth inhibitory assays. Seven out of the 20 compounds screened had 50% inhibitory concentration (IC_50_) below 500 nM. The most active among the selected compounds was MMV006087 (average IC_50_ of 30.79 nM). Variations in the potency of the Malaria Box Compounds were observed between *P. falciparum* clinical isolates and Dd2 strain. We also investigated the sensitivity of the clinical isolates to chloroquine and artesunate. The N093 clinical isolate was found to be resistant to chloroquine but showed high sensitivity to artesunate.

The results underscore the importance of including clinical isolates with different drug-resistant backgrounds, in addition to laboratory strains, in validating potential compounds during antimalarial compound screening programs.

## Highlights

• MMV006087, MMV085203, MMV008956 and MMV665977 were most potent compounds in this study.
• MMV006087 was the best compound in this study.
• N093 clinical isolate harboured K76T mutation and was sensitive to artesunate but resistant to chloroquine.
• Malaria Box Compounds have varied potency in clinical isolates and laboratory strains of *P. falciparum*.

## Graphical abstract

**Figure:**
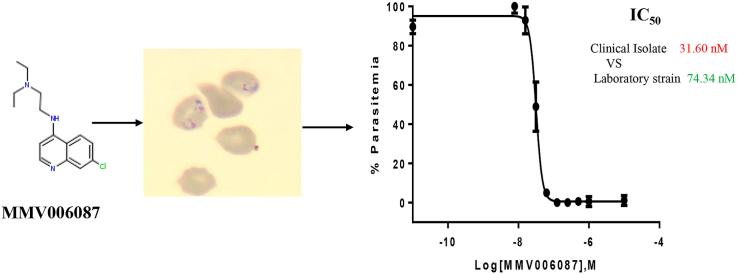

## 1 Introduction

*Plasmodium falciparum* related malaria is still a major threat to health systems in resource-limited settings. Since 2010, the global malaria incidence has declined by about 21%, however, malaria remains a major global health problem. An estimated 429,000 deaths were recorded in 2015 (WHO, 2016). About 90% of the global malaria-related deaths occurred in WHO Africa region with about 70% of these deaths occurring in children under five years (WHO, 2016). The global efforts to eliminate malaria over the past decades have largely been hampered by the development of insecticide and drug resistance by mosquitoes and *Plasmodium* respectively (Feachem et al., 2010, Ranson et al., 2009).

The emergence of resistant parasites to antimalarial drugs such as chloroquine (Moore and Lanier, 1961, Payne, 1987), sulfadoxine and pyrimethamine (Hurwitz, 1981) over the years, led to the recommendation for the use of artemisinin and artemisinin-based combination therapies (ACT), as first-line drugs for the treatment of malaria in all endemic regions (Smithuis et al., 2004, Valecha et al., 2010, WHO, 2001). Although there was a recent report *P. falciparum* isolate that was resistant to artemisinin in Equatorial Guinea (Lu et al., 2017), resistance to ACTs is still not wide-spread in Africa (WHO, 2016). Nevertheless, the recent emergence of ACT resistant *Plasmodium falciparum* strains in South-East Asia (Ashley et al., 2014, Dondorp et al., 2009, Noedl et al., 2008, Yeung et al., 2009), calls for new sets of antimalarial drugs with novel mechanisms of action.

Another important consideration is the lack of compliance to antimalarial drugs on the part of patients resulting in inadequate treatments, which may lead to selection and transmission of resistant parasites (White et al., 2009). The need for single dose drugs to promote patient compliance, with less ability to drive the development of resistance is therefore critical (Alonso et al., 2011). This not only offers an advantage of easy patient compliance, but also makes treatment less expensive (Burrows et al., 2013). In addition to possessing a long acting effect against drug resistant malaria parasites, such a drug should be able to reduce the parasite burden in asymptomatic individuals who serve as reservoirs for malaria transmission (Alonso et al., 2011, Diagana, 2015). Some recent studies have found a few antimalarial compounds with multi-stage activity (Baragana et al., 2015, Fidock, 2016, Kato et al., 2016), but there is the need to increase the repertoire of such compounds to provide the foundation for development of new drugs.

A number of drug screening studies have identified potent antimalarial compounds from the Malaria Box (Spangenberg et al., 2013) against laboratory-adapted strains of *P. falciparum* (Fong et al., 2015, Lucantoni et al., 2013, Van Voorhis et al., 2016). However, not much is known of the activity of these compounds against clinical isolates. In this study, *in vitro* drug susceptibility screening and polymerase chain reaction-restriction fragment length polymorphism (PCR-RFLP) genotyping analysis were adapted to confirm the potency of twenty selected Malaria Box Compounds using *P*. *falciparum* clinical isolates from Ghana. The compounds selected were based on their high potency against *P. falciparum* laboratory strains that was previously established using the entire Malaria Box library (Creek et al., 2016, Tiwari et al., 2016, Van Voorhis et al., 2016). The isolates were also screened against chloroquine and artesunate in defining their relative susceptibility to the different compounds. In addition, PCR-RFLP was used to investigate established drug associated mutations in four genes; *P. falciparum* chloroquine resistance transporter (*pfcrt*), *P. falciparum* multidrug resistance gene 1 (*pfmdr1*), *P. falciparum* dihydrofolate reductase gene (*pfdhfr*) and *P. falciparum* dihydropteroate synthase gene (*pfdhps*), known to mediate and/or modulate resistance to standard antimalarials.

## 2 Materials and methods

### 2.1 Clinical isolates

Four clinical isolates (K239, N093, A156 and A160) were randomly selected from archived samples from three endemic areas in Ghana, with different transmission intensities (Accra-low endemicity area, Kintampo-holoendemic area and Navrongo-hyperendemic area). The samples were collected as part of an ongoing study on erythrocyte invasion mechanisms (EIM). The samples were cryopreserved in liquid nitrogen immediately upon arrival from the field. The selected cryopreserved parasites were thawed and cultured with human blood group O^+^ erythrocytes using standard methods (Trager and Jensen, 1976) with slight modifications. *P. falciparum* clinical isolates were cultured to >5% (approximately 10 cycles) parasitemia of ring stage parasites. Using 5% Sorbitol treatment, a synchronized culture of ring-stage parasites (Lambros and Vanderberg, 1979) was obtained and diluted to 1% parasitemia in 2% haematocrit for the growth inhibition assays.

### 2.2 *In vitro* drug susceptibility assay

The library of 400 Malaria Box Compounds was provided by Medicines for Malaria Venture (MMV) at concentrations of 10 mM in dimethyl sulfoxide (DMSO) in 96-well microtiter plates (Spangenberg et al., 2013). A total of 20 compounds out of the 400 Malaria Box Compounds were selected for this study. The Malaria Box Compounds were serially diluted and screened against the clinical isolates at a concentration ranging from 0.064 nM to 25 μM. All the growth inhibition assays were set up in triplicate wells at final well volumes of 100 μL consisting of 10 μL of the test compound and 90 μL parasite culture at 1% parasitemia and 2% haematocrit in a 96-well flat bottom plate. The assay was incubated at 37 °C for 48 h. RPMI containing 0.25% DMSO was used as negative control whilst uninfected erythrocytes at 2% haematocrit were used as background control. After 48 h, 80 μL of the supernatant was taken out and replaced with 80 μL of SYBR Green I (Invitrogen, USA) stain, which was used to differentiate infected erythrocytes from uninfected erythrocytes by flow cytometry analysis. The plates were incubated in the dark with the SYBR Green I stain for 30 min prior to flow cytometry analysis.

The parasitemia corresponding to each culture well was quantified as previously described (Wirjanata et al., 2015) with modifications using BD FACS LSRFortessa™ X-20 flow cytometer with the BD FACSDiva Software (v8.0.1). The forward and side scatter parameters were used to gate erythrocytes and exclude debris. Photo multiplier tube (PMT) voltages of 200, 250 and 300 V were set for the forward scatter, side scatter and SYBR Green I, respectively. A dilution of 1:50 comprising 10 μL of packed erythrocytes to 490 μL of sheath fluid (BD Bioscience, USA) was used in the flow cytometry analysis. SYBR Green I positive erythrocytes corresponding with infected erythrocytes were used to determine the mean parasitemia levels. Data from 50,000 cells per well were recorded for all assays. The sensitivity of the clinical isolates to chloroquine and artesunate was also evaluated. Stock solutions of 10 mM chloroquine solution was prepared and used to screen against the four clinical isolates at final well concentrations between 0.0064 nM and 25 μM. The negative control was sterile distilled water in place of chloroquine. Artesunate stock of 1 mM artesunate (Sigma-Aldrich, USA) were similarly prepared in DMSO and tested at final well concentrations between 0.00248 nM and 32 nM. A 0.1% DMSO in RPMI was used in the negative control wells.

### 2.3 Determination of the molecular markers of drug resistance

*P. falciparum* genomic DNA was extracted from the clinical isolates (N093, A156, A160 and K239) using the QIAamp blood midi kit (QIAGEN, USA) and stored at −20 °C. Each of the isolates was analyzed for the putative point mutations that have been shown to mediate antimalarial drug resistance in the *pfcrt* (**K**76**T**), *pfmdr1* (**N**86**Y** and **Y**184**F**), *pfdhfr (***N**51**I**, **C**59**R** and **S**108**N**) and *pfdhps* (**A**437**G**) genes. Regions flanking these point mutations were amplified by polymerase chain reaction (PCR) using previously reported primer sets (Djimde et al., 2001, Duraisingh et al., 1998). One microlitre of the outer PCR products was used as template DNA in the nested PCR. Both the outer and the nested PCRs were set at a final volume of 25 μL containing 1X of Maxima Hot Start Green PCR master mix (Thermo Scientific, USA) and primers at a final concentration of 250 nM. Five microliters of the nested PCR products were run on ethidium bromide-stained 2% agarose gels and then viewed with the Amersham Imager 600 (General Electric Healthcare Life Sciences, USA). Five microliters of the remaining products were digested with restriction enzymes (New England BioLabs, USA) specific for the mutations, in a total volume of 15 μL. The restriction digest products were run on ethidium bromide-stained 2% agarose gels and visualized using the Amersham Imager 600. Purified *P. falciparum* genomic DNA from laboratory-adapted strains (Dd2, 3D7, K1, W2, FCR3 and 7G8) were used as controls.

### 2.4 Data analysis

The data from the flow cytometry analysis was first formatted in Microsoft Excel by subtracting the background fluorescence from all the data. GraphPad Prism (Version 6.01) was then used to generate sigmoidal dose-response curves by fitting a non-linear regression curve to the data. The 50% inhibitory concentration (IC_50_) values were then estimated from the dose response curves. Each data point on the dose-response is presented as the mean ± (SEM) of two experiments set up in triplicate.

### 2.5 Ethics

The studies were approved by the ethics committees of the Ghana Health Service, Navrongo Health Research Centre, Kintampo Health Research Centre, and Noguchi Memorial Institute for Medical Research, University of Ghana, Legon. All samples were collected after obtaining written informed consent from the parents/guardians of participating children who were aged ≤10 years. For children older than 10 years, additional assent was obtained from the donor, following receipt of parental consent. Blood used in this study for culturing was obtained from donors with informed consent.

## 3 Results

### 3.1 Determining the potency of Malaria Box Compounds against clinical and laboratory isolates of *P. falciparum*

**Fig. 1:**
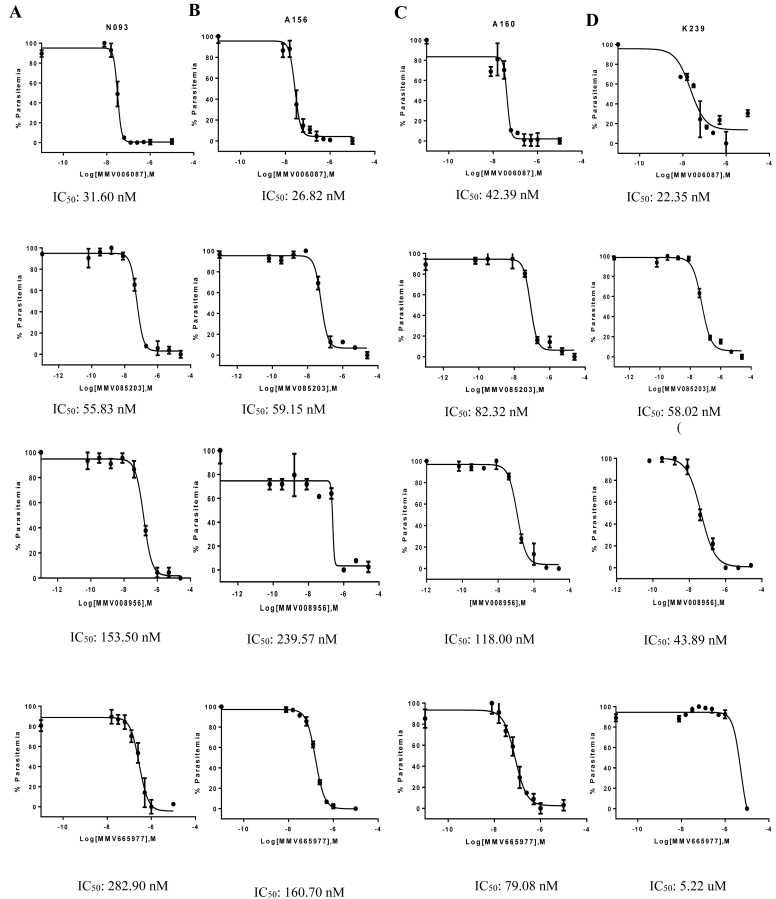
Dose-response curves of the four most potent Malaria box compounds against four clinical isolates of *P. falciparum*. Panel A–D are dose-response curves showing the response of clinical isolates (A) N093, (B) A156, (C) A160 and (D) K239 to MMV006087, MMV085203, MMV008956 and MMV665977 at concentrations from 0.064 nM to 25 μM. Each data point represents the mean ± SEM (n = 3). The plot shows percentage parasitemia against the log of the concentration of the compound. MMV006087 was the most potent compound followed by MM085203 and these two compounds had IC_50_ values less than 100 nM.

**Fig. 2:**
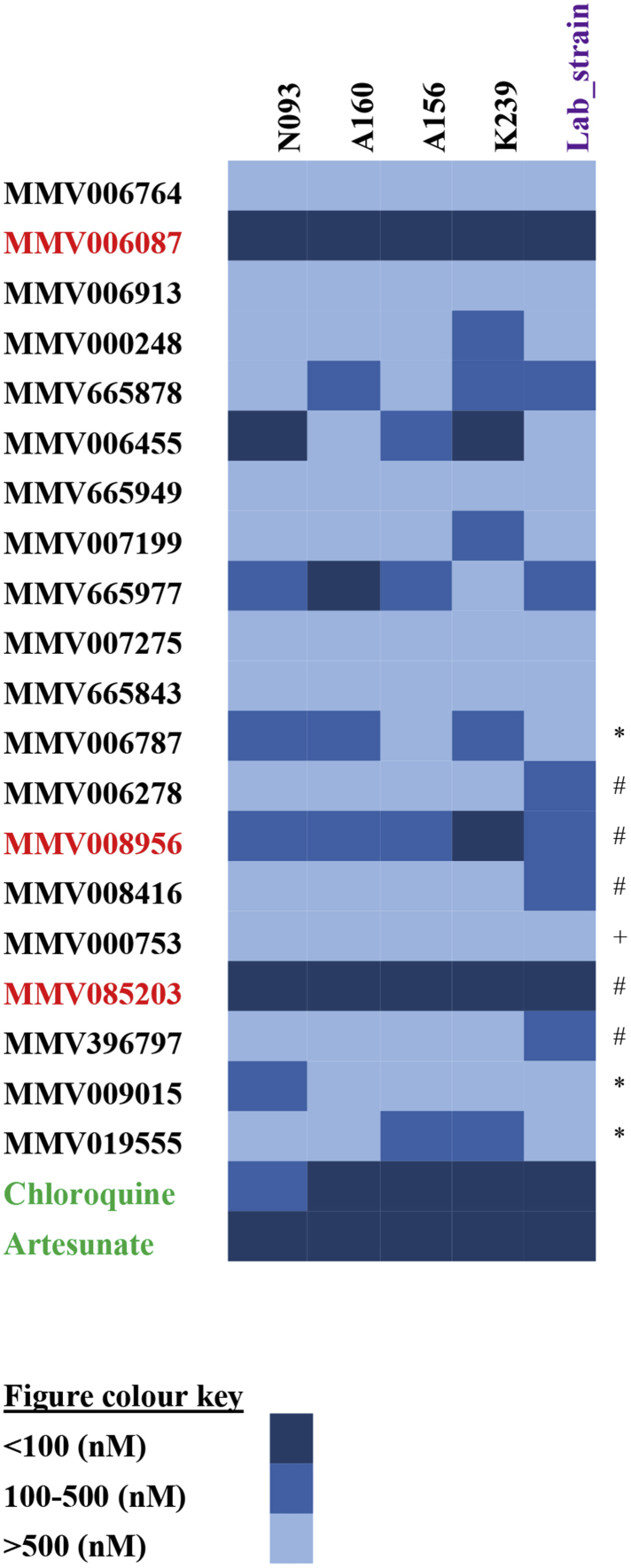
A heatmap showing the IC_50_ values for the 20 Malaria Box compounds against the four clinical isolates and laboratory strains. MMV006087 and MMV085203 were the two most potent compounds against all the four clinical isolates in the screen, with IC_50_ values below 100 nM. The last column with symbols (*, # and +) are IC_50_ values reported for Dd2, 3D7 and C235 respectively, from previous studies on these compounds (Fong et al., 2015, Lehane et al., 2014, Tiwari et al., 2016, Van Voorhis et al., 2016). Laboratory strains without symbols are IC_50_ values for Dd2 strains observed in this current study. Artesunate and chloroquine (highlighted green) were the standard antimalarial compounds used in this study. Artesunate was more effective against the clinical isolates compared to chloroquine. N093 was resistant to chloroquine but showed high sensitivity to artesunate. (For interpretation of the references to colour in this figure legend, the reader is referred to the web version of this article.)

A subset of 20 Malaria Box Compounds (Table 1) that have been found to be potent against laboratory strains of *P. falciparum* (Fong et al., 2015, Tiwari et al., 2016, Van Voorhis et al., 2016) were selected and evaluated against four clinical isolates of *P. falciparum* (N093, A160, A156 and K239). Out of the 20 compounds screened, the dose response curves of the four most potent compounds to all the four clinical isolates have been shown (Fig. 1). In total, seven compounds had IC_50_ values below 500 nM, six compounds had IC_50_ values between 500 nM and 1 μM and the remaining seven had IC_50_ values greater than 1 μM (Fig. 2). Considering compounds with IC_50_ values below 500 nM; three compounds (MMV008956, MMV085203 and MMV006087) were active against all four clinical isolates (Fig. 1, Fig. 2). Three compounds (MMV006787, MMV006455 and MMV665977) were active against three clinical isolates (Fig. 1, Fig. 2 and S1A) whilst two compounds (MMV019555 and MMV665878) were active against two clinical isolates (Fig. 2 and S1C). In addition, four compounds (MMV009015, MMV000248, MMV006764 and MMV007199) showed activity against one clinical isolate (Fig. 2, S1A to S1C). The rest of the compounds (MMV396797, MMV665843, MMV000753, MMV006764, MMV006913, MMV007275, MMV665949, MMV006278 and MMV008416) were less active against the clinical isolates with IC_50_ values greater than 500 nM (Fig. 2, S1B to S1D).

**Table 1:** **Summary of information on the 20 Malaria Box Compounds screened.** The structures and IC_50_ values for all the 20 compounds against the four clinical isolates and Dd2 are all shown.

1	MMV009015	MMV006278	MMV000753	MMV019555
			
	N093: 275.60 nM	N093: 1900 nM	N093: 1122 nM	N093: 1122 nM
	A156: 804.40 nM	A156: 18410 nM	A156: 769.60 nM	A156: 769.60 nM
	A160: 758.80 nM	A160: 9460 nM	A160: 811.10 nM	A160: 811.10nM
	K239: 1009 nM	K239: 4340 nM	K239: 940.70 nM	K239: 940.70 nM
2	MMV396797	MMV019555	MMV006787	MMV085203
			
	N093: 659.90 nM	N093: 3318 nM	N093: 190 nM	N093: 55.83 nM
	A156: 701.10 nM	A156: 280.70 nM	A156: 939.60 nM	A156: 59.15 nM
	A160: 835.80 nM	A160: 867.20 nM	A160: 418.60 nM	A160: 82.32 nM
	K239: 801.60 nM	K239: 399.90 nM	K239: 320.60 nM	K239: 58.02 nM
3	MMV006087	MMV665878	MMV006913	MMV008956
			
	N093: 31.60 nM	N093: 4164 nM	N093: 1.844 nM	N093: 153.50 nM
	A156: 26.82 nM	A156: 8100 nM	A156: 2.71 nM	A156: 239.57 nM
	A160: 42.39 nM	A160: 212.50 nM	A160: 1.443 nM	A160: 118 nM
	K239: 22.35 nM	K239: 175.80 nM	K239: 1.25 nM	K239: 43.89 nM
	Dd2: 74.34 nM	Dd2: 187.80 nM	Dd2: 2317 nM	
4	MMV000248	MMV007275	MMV665843	MMV006455
			
	N093: 554.10 nM	N093: 9022 nM	N093: 1166 nM	N093: 19.02 nM
	A156: 716.10 nM	A156: 6810 nM	A156: 776.90 nM	A156: 240.60 nM
	A160: 759.80 nM	A160: 3256 nM	A160: 713.50 nM	A160: 611.30 nM
	K239: 327.50 nM	K239: 5291 nM	K239: 921.20 nM	K239: 98.59 nM
	Dd2: 1119 nM	Dd2: 7508 nM	Dd2: 21930 nM	Dd2: 855.10 nM
5	MMV665949	MMV665977	MMV006764	MMV007199
			
	N093: 9764 nM	N093: 282.90 nM	N093: 1197 nM	N093: 1070 nM
	A156: 4138 nM	A156: 160.70 nM	A156: 435.5 nM	A156: 1849 nM
	A160: 8351 nM	A160: 79.08 nM	A160: 1334 nM	A160: 1089 nM
	K239: 3991 nM	K239: 5216 nM	K239: 993.80 nM	K239: 228.80 nM
	Dd2: 1309 nM	Dd2: 364.70 nM	Dd2: 7769 nM	Dd2: 2642 nM
	Artesunate	Chloroquine		
	
	N093: 2.69 nM	N093: 121.20 nM		
	A156: 12.68 nM	A156: 31.54 nM		
	A160: 3.09 nM	A160: 22.22 nM		
	K239: 4.78 nM	K239: 26.82 nM		

Summary of information (structures of the compounds and IC_50_ values) on the 20 Malaria Box compounds, artesunate and chloroquine screened against clinical isolates and Dd2-laboratory strain of *P. falciparum*.

The most potent compound in this screen was MMV006087, with an average IC_50_ of 30.79 nM, followed by MMV085203, with an average IC_50_ of 63.83 nM across the four clinical isolates (Fig. 2, Table 1). MMV006087 and MMV085203 generally showed comparable IC_50_ values to that of chloroquine in three of the clinical isolates (A156, A160 and K239), but exhibited about three-fold higher potency against N093 than chloroquine (Fig. 2 and S1E). Artesunate (Fig. 2) showed about eight-fold higher potency against the clinical isolates compared to MMV006087 and MMV085203 (Fig. 1).

As a reference, IC_50_ values for the 20 compounds against the laboratory strains of *P. falciparum* (either screened in our laboratory or from published studies) were compared to the IC_50_ values observed against the clinical isolates (Fig. 2). Thirteen of the 20 compounds (MMV006787, MMV008956, MMV000753, MMV006764, MMV006087, MMV006913, MMV000248, MMV006455, MMV007199, MMV665977, MMV007275, MMV009015 and MMV665843) were more potent against the clinical isolates compared to the laboratory-adapted strains (Fig. 2 and Table 1). The remaining seven compounds (MMV006278, MMV008416, MMV085203, MMV396797, MMV019555, MMV665878 and MMV665949) showed higher potency against laboratory strains of *P. falciparum* compared to the clinical isolates (Fig. 2). Also, compounds such as MMV665878 (Fig. S1C) and MMV665949 (Fig. S1D) showed higher potencies against some of the clinical isolates compared to Dd2.

### 3.2 Responses of clinical isolates to standard antimalarial drugs

To understand the drug sensitivity background of the clinical isolates used in this study, the clinical isolates were also screened against chloroquine and artesunate. The observed IC_50_ values were then compared with the standard IC_50_ values (Pradines et al., 2011) for both chloroquine and artesunate as well as the selected Malaria Box Compounds. The clinical isolate N093 was found to be resistant to chloroquine (IC_50_ > 100 nM), but was sensitive to artesunate (IC_50_ < 10.5 nM) (Table 1 and Fig. S1E). Relative to the Malaria Box compounds and chloroquine, artesunate was more potent against all the four clinical isolates with IC_50_ ranging from 2.69 to 12.68 nM (Fig. 2 and S1E). Artesunate showed less activity against A156 with an IC_50_ value of 12.68 nM which is about four-fold higher than the average for the other three isolates (Table 1 and Fig. S1E).

### 3.3 Drug resistance background of the clinical isolates

The PCR-RFLP analysis was used to determine molecular markers that are commonly used to detect antimalarial drug resistance. Four different genes with their corresponding mutations as have been implicated for drug resistance in *P. falciparum* were assessed. The respective mutations studied are; *pfcrt* codon 76 (Djimde et al., 2001), *pfmdr1* codons 86 and 184 (Foote et al., 1990), *pfdhps* codons 437 (Wang et al., 1997) and *pfdhfr* codons 51,108 and 59 (Peterson et al., 1988). Notably, N093 which exhibited resistance to chloroquine but was most sensitive to artesunate was found to harbour the *pfcrt* 76**T** mutant allele, whereas A156, A160 and K239 were found to contain the wild-type *pfcrt* **K**76 allele (Fig. S2 and Table 2). All the four clinical isolates had the mutant *pfmdr1* 184**F** allele (Fig. S2 and Table 2), whereas only N093 and K239 were found to contain the *pfmdr1* 86**Y** mutant allele (Fig. S2 and Table 2). For the *pfdhps*, all the clinical isolates had a mutant 437**G** allele with K239 showing mixed genotypes (Fig. S2 and Table 2). Two of the clinical isolates (A156 and N093) had the mutant *pfdhfr* 51**I** allele, whilst K239 was again found with mixed genotypes (Fig. S2 and Table 2).

**Table 2:** **Summary of polymorphisms in the *pfcrt, pfmdr1, pfdhps* and *pfdfr* genes in the clinical isolates**. N093 showed mutations that mediate chloroquine, multi-drug, sulfadoxine and pyrimethamine resistance respectively. A156 was sensitive to chloroquine, but had mutations that mediate multidrug resistance as well as sulfadoxine and pyrimethamine resistance. A160 was sensitive to both chloroquine and pyrimethamine but harboured mutations mediating multidrug and sulfadoxine resistance. K239, on the other hand was sensitive to chloroquine and sulfadoxine, but multi-drug resistant. K239 showed a mixed response to pyrimethamine and multidrug resistance.

Isolate ID	*Pfcrt*	*Pfmdr-1*		*Pfdhfr*			*Pfdhps*
	K76T	N86Y	Y184F	N51I	C59R	S108N	A437G
A156	W	W	M	M	M	M	M
A160	W	W	M	Y*	W	W	M
N093	M	M	M	M	M	M	M
K239	W	Y*	M	Y*	Y*	Y*	Y*

Summary of polymorphisms in the *pfcrt, pfmdr1, pfdhps* and *pfdfr* genes in the clinical isolates.

W = wildtype, M = mutant and Y* = Mixed infections.

## 4 Discussions

The ultimate target for any new antimalarial compounds will be clinical isolates. Studies have shown that clinical isolates from endemic areas have multiple clones of *P. falciparum* parasites, some of which might be drug resistant (Farnert et al., 2002, Ofosu-Okyere et al., 2001). Therefore, the use of clinical isolates of *P. falciparum* is necessary for drug discovery and susceptibility studies. Much screening activities have been conducted using the Malaria Box Compounds against laboratory strains of *P. falciparum*, but not much has been reported on the potency of these compounds against clinical isolates. It was therefore imperative to validate the most potent Malaria Box Compounds that were previously reported against *P. falciparum* clinical isolates from Ghana. In this study, we present evidence for the efficacy of Malaria Box Compounds against clinical isolates of *P. falciparum* from Ghana.

Our data shows that MMV008956, MMV085203, MMV006087 and MMV665977 were the most active compounds by IC_50_ values. However, MMV006087 was the most potent compound against all the isolates used in the screen. The high potency of this compound against the field isolates compared to the Dd2 strain implies a more susceptible phenotype in the clinical isolates, though they seem to have varied susceptibility signatures (Table 2). This compound has been shown to affect protein degradation pathways, similar to the action of chloroquine and piperaquine (Creek et al., 2016), whilst exhibiting fast-killing activity similar to that of artemisinin (Van Voorhis et al., 2016). MMV006087 has also been found to be active against early ring stage *P. falciparum* parasites as well as gametocytes (Van Voorhis et al., 2016). These unique properties of MMV006087 prioritize this compound as a suitable antimalarial candidate against blood stage and transmission stage *P. falciparum* parasites.

The glutathione and thioredoxin systems may provide a way for malaria parasites to maintain redox homeostasis and antioxidant defense, considering that *P. falciparum* lacks glutathione peroxidase and catalase (Jortzik and Becker, 2012). Studies using genetic and chemical tools have shown that. *P. falciparum* thioredoxin reductase (P*f*TrxR) is a target necessary for the survival of the parasite (Muller, 2003). In a study by Tiwari et al. MMV085203 and MMV008956 were both found to target P*f*TrxR, but MMVV085203 was more potent at inhibiting this target than MMV008956. Based on the IC_50_ values observed in this study, MMV085203 was also found to be more potent at killing the parasite than MMV008956. The efficient killing with lower IC_50_ values of these two compounds indicates the possible essentiality of the proteins involved in the antioxidant defense system (Arner and Holmgren, 2000, Gilberger et al., 2000). The compound MV000753, an inhibitor of hemozoin formation (Fong et al., 2015), was found in this study to be more effective against the clinical isolates than was reported for D6; a chloroquine sensitive strain, and C235, a multi-drug resistant strain, (Fong et al., 2015). These differences in potency observed in both the clinical isolates and laboratory-adapted strains suggest the need for thorough screening with clinical isolates in antimalarial drug discovery studies.

Studies have shown that the presence of mutations in the *pfcrt* and *pfmdr1* genes of *P. falciparum* parasites not only decrease their susceptibility to quinine and halofantrine, but increase their sensitivity to artemisinin and lumefantrine (Gresty et al., 2014, Reed et al., 2000, Sidhu et al., 2005). In this current study, the N093 clinical isolate was the only parasite that was found to have polymorphisms in both the *pfmdr1* and *pfcrt* genes. The N093 clinical isolate was also found to be the only isolate that was very sensitive to artesunate but resistant to chloroquine in this study. It therefore suggests that the presence of SNPs in *pfmdr1* and *pfcrt* genes might play a key role in modulating the sensitivity of this isolate to artesunate, and its resistance to chloroquine, as was observed by other studies (Gresty et al., 2014, Reed et al., 2000, Sidhu et al., 2005).

## 5 Conclusion

The findings suggest that the Malaria Box Compounds have varied activities against different clinical isolates. It has been shown that of all the compounds screened, MMV006087 had the best IC_50_ across the different clinical isolates, with N093 being a chloroquine resistant parasite. From the data, having multiclonal populations also presents different sensitivity as was observed for K239. The data shows MMV006087, MMV085203 and MMV008956 compounds as very potent against the four Ghanaian clinical isolates suggesting they are very promising compounds and further analysis needs to be done to validate their suitability as candidates in antimalarial drug development.

## Conflict of interest

The authors declare no conflict of interest.

## References

[bib1] Alonso P.L., Brown G., Arevalo-Herrera M., Binka F., Chitnis C., Collins F., Doumbo O.K., Greenwood B., Hall B.F., Levine M.M., Mendis K., Newman R.D., Plowe C.V., Rodriguez M.H., Sinden R., Slutsker L., Tanner M. (2011). A research agenda to underpin malaria eradication. PLoS Med..

[bib2] Arner E.S., Holmgren A. (2000). Physiological functions of thioredoxin and thioredoxin reductase. Eur. J. Biochem..

[bib3] Ashley E.A., Dhorda M., Fairhurst R.M., Amaratunga C., Lim P., Suon S., Sreng S., Anderson J.M., Mao S., Sam B., Sopha C., Chuor C.M., Nguon C., Sovannaroth S., Pukrittayakamee S., Jittamala P., Chotivanich K., Chutasmit K., Suchatsoonthorn C., Runcharoen R., Hien T.T., Thuy-Nhien N.T., Thanh N.V., Phu N.H., Htut Y., Han K.T., Aye K.H., Mokuolu O.A., Olaosebikan R.R., Folaranmi O.O., Mayxay M., Khanthavong M., Hongvanthong B., Newton P.N., Onyamboko M.A., Fanello C.I., Tshefu A.K., Mishra N., Valecha N., Phyo A.P., Nosten F., Yi P., Tripura R., Borrmann S., Bashraheil M., Peshu J., Faiz M.A., Ghose A., Hossain M.A., Samad R., Rahman M.R., Hasan M.M., Islam A., Miotto O., Amato R., MacInnis B., Stalker J., Kwiatkowski D.P., Bozdech Z., Jeeyapant A., Cheah P.Y., Sakulthaew T., Chalk J., Intharabut B., Silamut K., Lee S.J., Vihokhern B., Kunasol C., Imwong M., Tarning J., Taylor W.J., Yeung S., Woodrow C.J., Flegg J.A., Das D., Smith J., Venkatesan M., Plowe C.V., Stepniewska K., Guerin P.J., Dondorp A.M., Day N.P., White N.J., Tracking Resistance to Artemisinin, C (2014). Spread of artemisinin resistance in Plasmodium falciparum malaria. N. Engl. J. Med..

[bib4] Baragana B., Hallyburton I., Lee M.C.S., Norcross N.R., Grimaldi R., Otto T.D., Proto W.R., Blagborough A.M., Meister S., Wirjanata G., Ruecker A., Upton L.M., Abraham T.S., Almeida M.J., Pradhan A., Porzelle A., Martinez M.S., Bolscher J.M., Woodland A., Norval S., Zuccotto F., Thomas J., Simeons F., Stojanovski L., Osuna-Cabello M., Brock P.M., Churcher T.S., Sala K.A., Zakutansky S.E., Jimenez-Diaz M.B., Sanz L.M., Riley J., Basak R., Campbell M., Avery V.M., Sauerwein R.W., Dechering K.J., Noviyanti R., Campo B., Frearson J.A., Angulo-Barturen I., Ferrer-Bazaga S., Gamo F.J., Wyatt P.G., Leroy D., Siegl P., Delves M.J., Kyle D.E., Wittlin S., Marfurt J., Price R.N., Sinden R.E., Winzeler E.A., Charman S.A., Bebrevska L., Gray D.W., Campbell S., Fairlamb A.H., Willis P.A., Rayner J.C., Fidock D.A., Read K.D., Gilbert I.H. (2015). A novel multiple-stage antimalarial agent that inhibits protein synthesis. Nature.

[bib5] Burrows J.N., van Huijsduijnen R.H., Mohrle J.J., Oeuvray C., Wells T.N. (2013). Designing the next generation of medicines for malaria control and eradication. Malar. J..

[bib6] Creek D.J., Chua H.H., Cobbold S.A., Nijagal B., Macrae J.I., Dickerman B.K., Gilson P.R., Ralph S.A., McConville M.J. (2016). Metabolomics-based screening of the Malaria Box reveals both novel and established mechanisms of action. Antimicrob. Agents Chemother..

[bib7] Diagana T.T. (2015). Supporting malaria elimination with 21st century antimalarial agent drug discovery. Drug Discov. today.

[bib8] Djimde A., Doumbo O.K., Cortese J.F., Kayentao K., Doumbo S., Diourte Y., Coulibaly D., Dicko A., Su X.Z., Nomura T., Fidock D.A., Wellems T.E., Plowe C.V. (2001). A molecular marker for chloroquine-resistant falciparum malaria. N. Engl. J. Med..

[bib9] Dondorp A.M., Nosten F., Yi P., Das D., Phyo A.P., Tarning J., Lwin K.M., Ariey F., Hanpithakpong W., Lee S.J., Ringwald P., Silamut K., Imwong M., Chotivanich K., Lim P., Herdman T., An S.S., Yeung S., Singhasivanon P., Day N.P., Lindegardh N., Socheat D., White N.J. (2009). Artemisinin resistance in Plasmodium falciparum malaria. N. Engl. J. Med..

[bib10] Duraisingh M.T., Curtis J., Warhurst D.C. (1998). Plasmodium falciparum: detection of polymorphisms in the dihydrofolate reductase and dihydropteroate synthetase genes by PCR and restriction digestion. Exp. Parasitol..

[bib11] Farnert A., Tengstam K., Palme I.B., Bronner U., Lebbad M., Swedberg G., Bjorkman A. (2002). Polyclonal Plasmodium falciparum malaria in travelers and selection of antifolate mutations after proguanil prophylaxis. Am. J. Trop. Med. Hyg..

[bib12] Feachem R.G., Phillips A.A., Targett G.A., Snow R.W. (2010). Call to action: priorities for malaria elimination. Lancet.

[bib13] Fidock D.A. (2016). Drug discovery: chemical diversity targets malaria. Nature.

[bib14] Fong K.Y., Sandlin R.D., Wright D.W. (2015). Identification of beta-hematin inhibitors in the MMV Malaria Box. International journal for parasitology. Drugs drug Resist..

[bib15] Foote S.J., Kyle D.E., Martin R.K., Oduola A.M., Forsyth K., Kemp D.J., Cowman A.F. (1990). Several alleles of the multidrug-resistance gene are closely linked to chloroquine resistance in Plasmodium falciparum. Nature.

[bib16] Gilberger T.W., Schirmer R.H., Walter R.D., Muller S. (2000). Deletion of the parasite-specific insertions and mutation of the catalytic triad in glutathione reductase from chloroquine-sensitive Plasmodium falciparum 3D7. Mol. Biochem. Parasitol..

[bib17] Gresty K.J., Gray K.-A., Bobogare A., Taleo G., Hii J., Wini L., Cheng Q., Waters N.C. (2014). Genetic mutations in pfcrt and pfmdr1 at the time of artemisinin combination therapy introduction in South Pacific islands of Vanuatu and Solomon Islands. Malar. J..

[bib18] Hurwitz E. (1981). Resistance of plasmodium falciparum malaria to sulfadoxine-pyrimethamine ('Fansidar') in a refugee camp in Thailand. Lancet.

[bib19] Jortzik E., Becker K. (2012). Thioredoxin and glutathione systems in Plasmodium falciparum. Int. J. Med. Microbiol. IJMM.

[bib20] Kato N., Comer E., Sakata-Kato T., Sharma A., Sharma M., Maetani M., Bastien J., Brancucci N.M., Bittker J.A., Corey V., Clarke D., Derbyshire E.R., Dornan G.L., Duffy S., Eckley S., Itoe M.A., Koolen K.M., Lewis T.A., Lui P.S., Lukens A.K., Lund E., March S., Meibalan E., Meier B.C., McPhail J.A., Mitasev B., Moss E.L., Sayes M., Van Gessel Y., Wawer M.J., Yoshinaga T., Zeeman A.M., Avery V.M., Bhatia S.N., Burke J.E., Catteruccia F., Clardy J.C., Clemons P.A., Dechering K.J., Duvall J.R., Foley M.A., Gusovsky F., Kocken C.H., Marti M., Morningstar M.L., Munoz B., Neafsey D.E., Sharma A., Winzeler E.A., Wirth D.F., Scherer C.A., Schreiber S.L. (2016). Diversity-oriented synthesis yields novel multistage antimalarial inhibitors. Nature.

[bib21] Lambros C., Vanderberg J.P. (1979). Synchronization of Plasmodium falciparum erythrocytic stages in culture. J. Parasitol..

[bib22] Lehane A.M., Ridgway M.C., Baker E., Kirk K. (2014). Diverse chemotypes disrupt ion homeostasis in the Malaria parasite. Mol. Microbiol..

[bib23] Lu F., Culleton R., Zhang M., Ramaprasad A., von Seidlein L., Zhou H., Zhu G., Tang J., Liu Y., Wang W., Cao Y., Xu S., Gu Y., Li J., Zhang C., Gao Q., Menard D., Pain A., Yang H., Zhang Q., Cao J. (2017). Emergence of indigenous artemisinin-resistant plasmodium falciparum in Africa. N. Engl. J. Med..

[bib24] Lucantoni L., Duffy S., Adjalley S.H., Fidock D.A., Avery V.M. (2013). Identification of MMV malaria box inhibitors of plasmodium falciparum early-stage gametocytes using a luciferase-based high-throughput assay. Antimicrob. Agents Chemother..

[bib25] Moore D.V., Lanier J.E. (1961). Observations on two plasmodium falciparum infections with an abnormal response to chloroquine. Am. J. Trop. Med. Hyg..

[bib26] Muller S. (2003). Thioredoxin reductase and glutathione synthesis in Plasmodium falciparum. Redox Rep. Commun. free Radic. Res..

[bib27] Noedl H., Se Y., Schaecher K., Smith B.L., Socheat D., Fukuda M.M. (2008). Evidence of artemisinin-resistant malaria in western Cambodia. N. Engl. J. Med..

[bib28] Ofosu-Okyere A., Mackinnon Mj Fau - Sowa M.P., Sowa Mp Fau - Koram K.A., Koram Ka Fau - Nkrumah F., Nkrumah F Fau - Osei Y.D., Osei Yd Fau - Hill W.G., Hill Wg Fau - Wilson M.D., Wilson Md Fau - Arnot D.E., Arnot D.E. (2001). Novel Plasmodium Falciparum Clones and Rising Clone Multiplicities Are Associated with the Increase in Malaria Morbidity in Ghanaian Children during the Transition into the High Transmission Season.

[bib29] Payne D. (1987). Spread of chloroquine resistance in Plasmodium falciparum. Parasitol. Today.

[bib30] Peterson D.S., Walliker D., Wellems T.E. (1988). Evidence that a point mutation in dihydrofolate reductase-thymidylate synthase confers resistance to pyrimethamine in falciparum malaria. Proc. Natl. Acad. Sci. U. S. A.

[bib31] Pradines B., Bertaux L., Pomares C., Delaunay P., Marty P. (2011). Reduced in vitro susceptibility to artemisinin derivatives associated with multi-resistance in a traveller returning from South-East Asia. Malar. J..

[bib32] Ranson H., Abdallah H., Badolo A., Guelbeogo W.M., Kerah-Hinzoumbe C., Yangalbe-Kalnone E., Sagnon N., Simard F., Coetzee M. (2009). Insecticide resistance in Anopheles gambiae: data from the first year of a multi-country study highlight the extent of the problem. Malar. J..

[bib33] Reed M.B., Saliba K.J., Caruana S.R., Kirk K., Cowman A.F. (2000). Pgh1 modulates sensitivity and resistance to multiple antimalarials in Plasmodium falciparum. Nature.

[bib34] Sidhu A.B., Valderramos S.G., Fidock D.A. (2005). pfmdr1 mutations contribute to quinine resistance and enhance mefloquine and artemisinin sensitivity in Plasmodium falciparum. Mol. Microbiol..

[bib35] Smithuis F., van der Broek I., Katterman N., Kyaw M.K., Brockman A., Lwin S., White N.J. (2004). Optimising operational use of artesunate-mefloquine: a randomised comparison of four treatment regimens. Trans. R. Soc. Trop. Med. Hyg..

[bib36] Spangenberg T., Burrows J.N., Kowalczyk P., McDonald S., Wells T.N., Willis P. (2013). The open access malaria box: a drug discovery catalyst for neglected diseases. PLoS One.

[bib37] Tiwari N.K., Reynolds P.J., Calderon A.I. (2016). Preliminary LC-MS based screening for inhibitors of plasmodium falciparum thioredoxin reductase (PfTrxR) among a set of antimalarials from the malaria box. Molecules.

[bib38] Trager W., Jensen J.B. (1976). Human malaria parasites in continuous culture. Science.

[bib39] Valecha N., Phyo A.P., Mayxay M., Newton P.N., Krudsood S., Keomany S., Khanthavong M., Pongvongsa T., Ruangveerayuth R., Uthaisil C., Ubben D., Duparc S., Bacchieri A., Corsi M., Rao B.H., Bhattacharya P.C., Dubhashi N., Ghosh S.K., Dev V., Kumar A., Pukrittayakamee S. (2010). An open-label, randomised study of dihydroartemisinin-piperaquine versus artesunate-mefloquine for falciparum malaria in Asia. PLoS One.

[bib40] Van Voorhis W.C., Adams J.H., Adelfio R., Ahyong V., Akabas M.H., Alano P., Alday A., Aleman Resto Y., Alsibaee A., Alzualde A., Andrews K.T., Avery S.V., Avery V.M., Ayong L., Baker M., Baker S., Ben Mamoun C., Bhatia S., Bickle Q., Bounaadja L., Bowling T., Bosch J., Boucher L.E., Boyom F.F., Brea J., Brennan M., Burton A., Caffrey C.R., Camarda G., Carrasquilla M., Carter D., Belen Cassera M., Chih-Chien Cheng K., Chindaudomsate W., Chubb A., Colon B.L., Colon-Lopez D.D., Corbett Y., Crowther G.J., Cowan N., D'Alessandro S., Le Dang N., Delves M., DeRisi J.L., Du A.Y., Duffy S., Abd El-Salam El-Sayed S., Ferdig M.T., Fernandez Robledo J.A., Fidock D.A., Florent I., Fokou P.V., Galstian A., Gamo F.J., Gokool S., Gold B., Golub T., Goldgof G.M., Guha R., Guiguemde W.A., Gural N., Guy R.K., Hansen M.A., Hanson K.K., Hemphill A., Hooft van Huijsduijnen R., Horii T., Horrocks P., Hughes T.B., Huston C., Igarashi I., Ingram-Sieber K., Itoe M.A., Jadhav A., Naranuntarat Jensen A., Jensen L.T., Jiang R.H., Kaiser A., Keiser J., Ketas T., Kicka S., Kim S., Kirk K., Kumar V.P., Kyle D.E., Lafuente M.J., Landfear S., Lee N., Lee S., Lehane A.M., Li F., Little D., Liu L., Llinas M., Loza M.I., Lubar A., Lucantoni L., Lucet I., Maes L., Mancama D., Mansour N.R., March S., McGowan S., Medina Vera I., Meister S., Mercer L., Mestres J., Mfopa A.N., Misra R.N., Moon S., Moore J.P., Morais Rodrigues da Costa F., Muller J., Muriana A., Nakazawa Hewitt S., Nare B., Nathan C., Narraidoo N., Nawaratna S., Ojo K.K., Ortiz D., Panic G., Papadatos G., Parapini S., Patra K., Pham N., Prats S., Plouffe D.M., Poulsen S.A., Pradhan A., Quevedo C., Quinn R.J., Rice C.A., Abdo Rizk M., Ruecker A., St Onge R., Salgado Ferreira R., Samra J., Robinett N.G., Schlecht U., Schmitt M., Silva Villela F., Silvestrini F., Sinden R., Smith D.A., Soldati T., Spitzmuller A., Stamm S.M., Sullivan D.J., Sullivan W., Suresh S., Suzuki B.M., Suzuki Y., Swamidass S.J., Taramelli D., Tchokouaha L.R., Theron A., Thomas D., Tonissen K.F., Townson S., Tripathi A.K., Trofimov V., Udenze K.O., Ullah I., Vallieres C., Vigil E., Vinetz J.M., Voong Vinh P., Vu H., Watanabe N.A., Weatherby K., White P.M., Wilks A.F., Winzeler E.A., Wojcik E., Wree M., Wu W., Yokoyama N., Zollo P.H., Abla N., Blasco B., Burrows J., Laleu B., Leroy D., Spangenberg T., Wells T., Willis P.A. (2016). Open source drug discovery with the malaria box compound collection for neglected diseases and beyond. PLoS Pathog..

[bib41] Wang P., Lee C.-S., Bayoumi R., Djimde A., Doumbo O., Swedberg G., Dao L.D., Mshinda H., Tanner M., Watkins W.M., Sims P.F.G., Hyde J.E. (1997). Resistance to antifolates in Plasmodium falciparum monitored by sequence analysis of dihydropteroate synthetase and dihydrofolate reductase alleles in a large number of field samples of diverse origins. Mol. Biochem. Parasitol..

[bib42] White N.J., Pongtavornpinyo W., Maude R.J., Saralamba S., Aguas R., Stepniewska K., Lee S.J., Dondorp A.M., White L.J., Day N.P. (2009). Hyperparasitaemia and low dosing are an important source of anti-malarial drug resistance. Malar. J..

[bib43] WHO (2001). WHO: Antimalarial Drug Combination Therapy. Report of a WHO Technical Consultation. http://www.who.int/malaria/publications/atoz/who_cds_rbm_2001_35/en/index.html.

[bib44] WHO (2016). World Malaria Report 2016.

[bib45] Wirjanata G., Sebayang B.F., Chalfein F., Prayoga, Handayuni I., Noviyanti R. (2015). Contrasting ex vivo efficacies of “reversed chloroquine” compounds in chloroquine-resistant Plasmodium falciparum and P. vivax isolates. Antimicrob. Agents Chemother..

[bib46] Yeung S., Socheat D., Moorthy V.S., Mills A.J. (2009). Artemisinin resistance on the Thai-Cambodian border. Lancet.

