# Supplementary material for: Antimalarial activity of Malaria Box Compounds against Plasmodium falciparum clinical isolates
Source: Int J Parasitol Drugs Drug Resist. 2017 Oct 16;7(3):399–406. doi: 10.1016/j.ijpddr.2017.10.005 (PMC5683671; doi:10.1016/j.ijpddr.2017.10.005)
Supplement: Online Data [file mmc1.docx]

**Figure S1A**

IC_50_: 98.59 nM

IC_50_: 240.60 nM

IC_50_: 19.02 nM

IC_50_: 611.30 nM

IC_50_: 190 nM

IC_50_: 939.60 nM

IC_50_: 418.60 nM

IC_50_:320.60 nM

IC_50_: 327.50 nM

IC_50_: 716.10 nM

IC_50_: 554.10 nM

IC_50_: 759.80 nM

IC_50_: 275.60 nM

IC_50_: 804.40 nM

IC_50_: 758.80 nM

IC_50_: 1009 nM

**A**

**D**

**C**

**B**

**Screening of four Malaria Box Compounds (MMV006455, MMV006787, MMV000248 and MMV009015) against four clinical isolates (N093, A156, A160 and K239) of *P. falciparum*.**

**Figure S1B**

IC_50_: 435.5 nM

IC_50_: 993.8 nM

IC_50_:1334 nM

IC_50_: 1197 nM

IC_50_: 940.70 nM

IC_50_:1122 nM

IC_50_: 811.10 nM

IC_50_: 769.60 nM

IC_50_: 921.20 nM

IC_50_:713.50 nM

IC_50_: 776.90 nM

IC_50_: 701.10 nM

IC_50_: 659.90 nM

IC_50_: 835.80 nM

IC_50_: 801.60 nM

IC_50_: 1166 nM

**N093**

**A156**

**A160**

**K239**

**A**

**B**

**C**

**D**

**Screening of four Malaria Box Compounds (MMV396797, MMV665843, MMV000753 and MMV006764) against four clinical isolates (N093, A156, A160 and K239) of *P. falciparum*.**

**Figure S1C**

IC_50_: 1849 nM

IC_50_: 1070 nM

IC_50_: 228.80 nM

IC_50_: 1089 nM

IC_50_: 3318 nM

IC_50_:280.70 nM

IC_50_: 867.20 nM

IC_50_: 399.90 nM

IC_50_0: 1443 nM

IC_50_: 1254 nM

IC_50_: 1844 nM

IC_50_: 2706 nM

IC_50_: 8100 nM

IC_50_: 175.80 nM

IC_50_: 4164 nM

IC_50_: 212.50 nM

**A**

**B**

**C**

**D**

**Screening of four Malaria Box Compounds (MMV007199, MMV019555, MMV006913 and MMV665878) against four clinical isolates (N093, A156, A160 and K239) of *P. falciparum*.**

**Figure S1D**

IC_50_: 1700 nM

Inactive

Inactive

IC_50_: 3530 nM

IC_50_:1900 nM

IC_50_:18410 nM

IC_50_:4340 nM

IC_50_: 9460 nM

IC_50_: 3991 nM

IC_50_0: 5138 nM

IC_50_: 9764 nM

IC_50_: 8351 nM

IC_50_: 9022 nM

IC_50_: 3256 nM

IC_50_: 6810 nM

IC_50_: 5291 nM

**A**

**B**

**C**

**D**

**Screening of four Malaria Box Compounds (MMV007275, MMV665949, MMV006278 and MMV008416) against four clinical isolates (N093, A156, A160 and K239) of *P. falciparum*.**

**Figure S1E**

**A**

**B**

**C**

**D**

N093

A156

A160

K239

IC_50_: 2.69 nM

IC_50_: 3.09 nM

IC_50_: 4.78 nM

IC_50_: 12.68 nM

IC_50_:121.20 nM

IC_50_:31.54 nM

IC_50_:22.22 nM

IC_50_: 26.82 nM

**Screening of the four clinical isolates of *P. falciparum* against chloroquine and artesunate**

**Figure S2**

**A**

64 bp

K239

N093

A160

A156

W2 (+)

NTC

LADDER

FCR3 (-)

100 bp

300 bp

500 bp

1000 bp

120 bp

218 bp


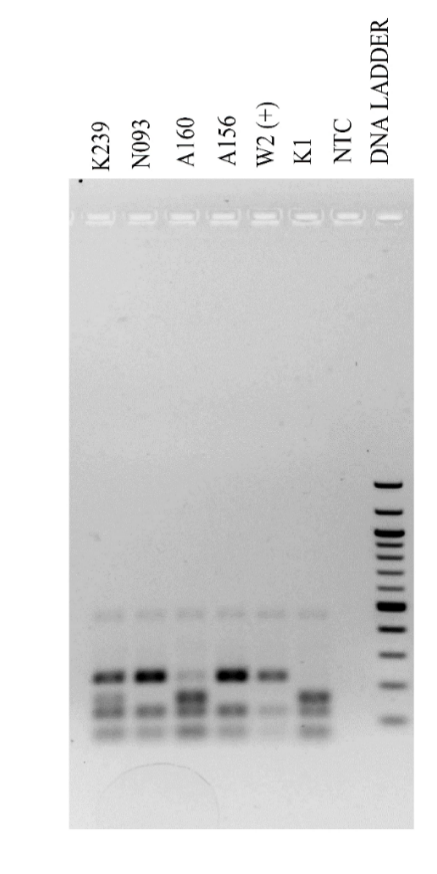


522 bp

190 bp

LADDER

NTC

K239

N093

A160

K1 (+)

FCR3 (-)

A160

K239

200 bp

100 bp

1000 bp

332 bp


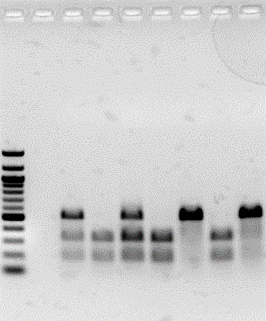


500 bp

**B**

N093

A160

A156

W2 (+)

LADDER

FCR3 (-)

K239

500 bp

200 bp

100 bp

1000 bp

189 bp

137 bp

**C**


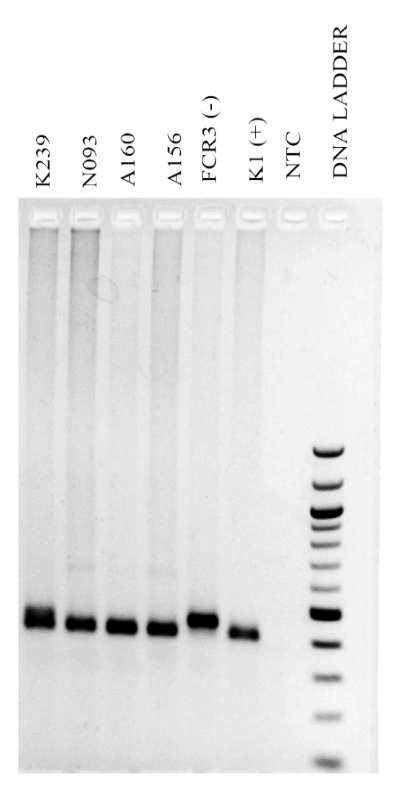


K239

N093

A160

A156

K1 (+)

NTC

LADDER

FCR3 (-)

100 bp

200 bp

500 bp

1000 bp

438 bp

404 bp

**D**

34 bp

134 bp

100 bp

200 bp

500 bp

1000 bp

K239

N093

A160

A156

3D7 (-)

Dd2 (+)

NTC

LADDER


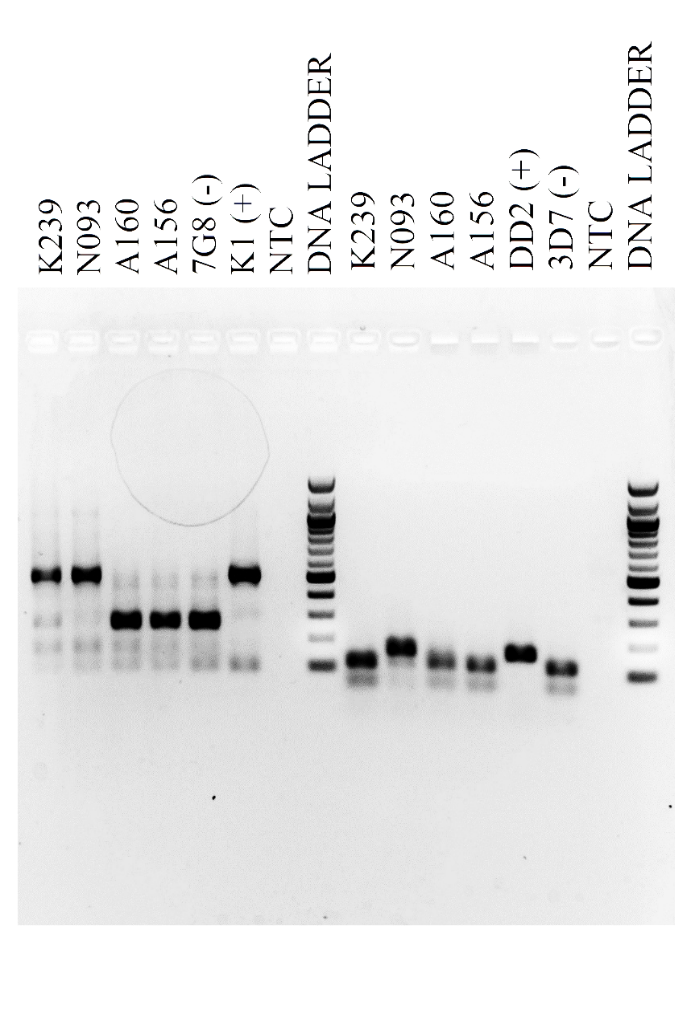


**E.**


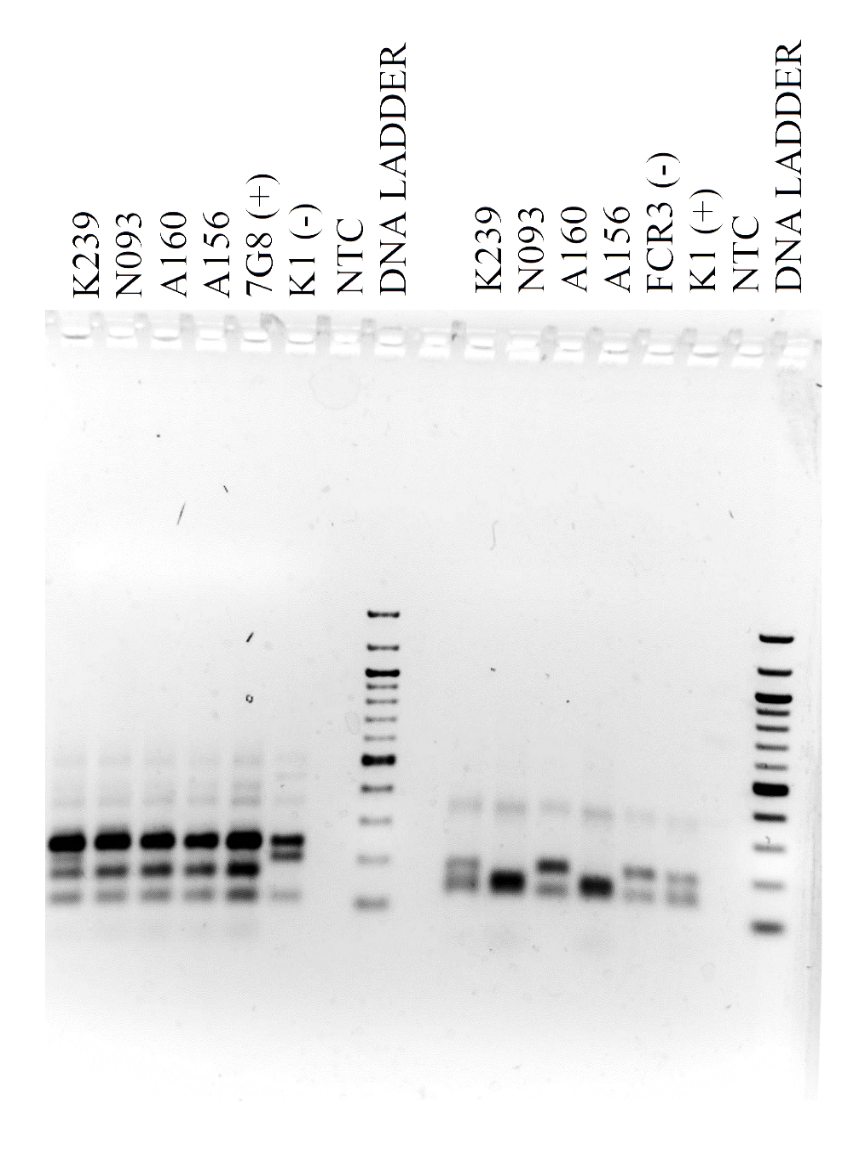


114 bp

242 bp

173 bp

100 bp

200 bp

500 bp

1000 bp

N093

A160

A156

K1 (-)

NTC

LADDER

7G8 (+)

**F**

K239

**G.**


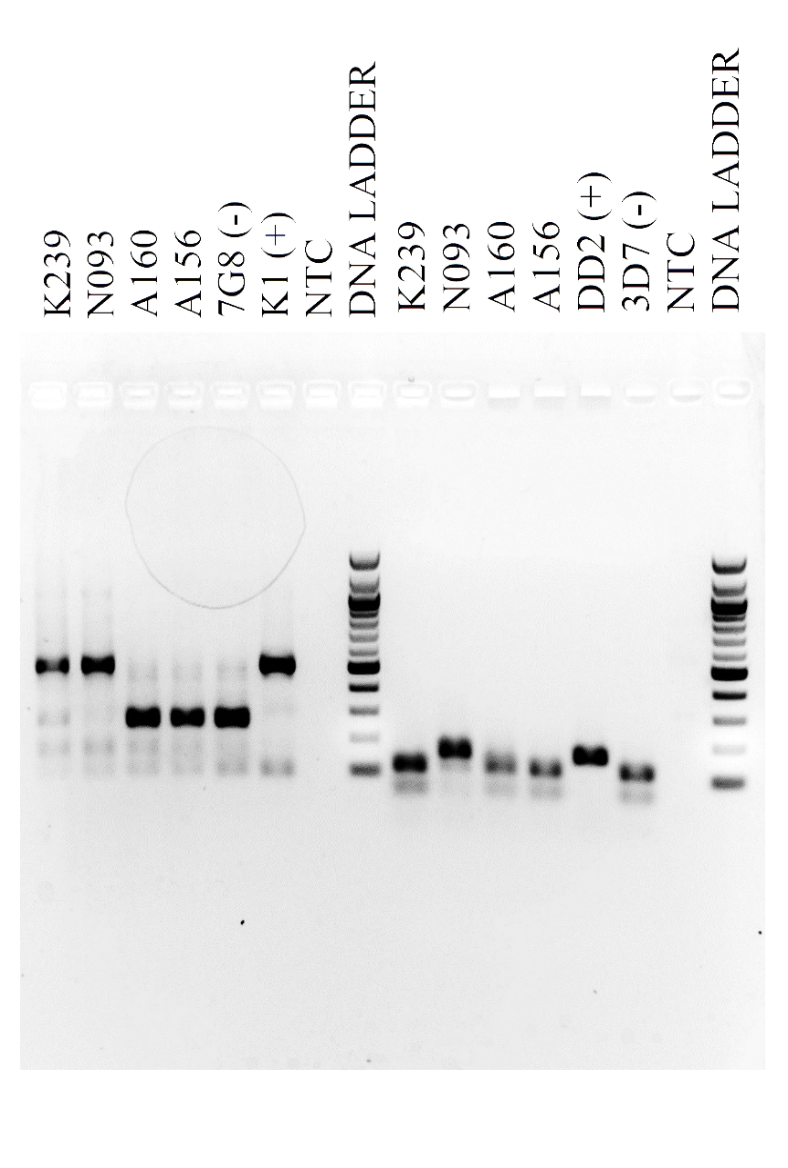


K239

N093

A160

A156

K1 (+)

NTC

LADDER

7G8 (-)

100 bp

200 bp

500 bp

1000 bp

249 bp, 256 bp

505 bp

**Identification of single nucleotide polymorphisms (SNPs) in the *pfdhfr* (A, B and C)*, pfdhps* (D)*, pfcrt* (E) and *pfmdr1* (F and G) genes of the clinical isolates.**
